# Optimization of SWCNT‐FET Biosensors by Aptamer Engineering and Toehold‐Mediated Strand Displacement

**DOI:** 10.1002/advs.75328

**Published:** 2026-04-16

**Authors:** Haosen Miao, Tingting Zheng, Houlin Yu, Gririraj Manoharan, Gustavo Sant'Anna, Nicholas Bedfrod, Jorge L. Chávez, Chang‐Seuk Lee, Matteo Palma

**Affiliations:** ^1^ Department of Chemistry Queen Mary University of London London UK; ^2^ School of Chemical Engineering University of New South Wales Sydney New South Wales Australia; ^3^ 711th Human Performance Wing Air Force Research Laboratory Ohio USA; ^4^ Department of Chemistry Seoul Women's University Seoul Republic of Korea

**Keywords:** aptamer, biosensors, cortisol, single‐walled carbon nanotubes, toehold‐mediated strand displacement

## Abstract

Aptamer‐based single‐walled carbon nanotube (SWCNT) field‐effect transistor (FET) biosensors provide a compact, label‐free, and sensitive platform for real‐time biomarker detection. To enhance device performance, we employed a truncated DNA aptamer (CSS.3_cut3) with improved conformational switching upon cortisol binding, boosting the sensitivity of SWCNT‐FET biosensors. We further optimized the functionalization using a toehold‐mediated strand displacement (TMSD) strategy to dynamically integrate the aptamer onto SWCNTs in solution, simplifying the fabrication process and increasing the sensitivity. Interestingly, introducing a partially complementary protection strand during TMSD assembly resulted in bistable, two‐level signal outputs upon cortisol sensing, contrasting with the gradual response of unprotected sensors. This effect likely stems from the competition between aptamer–target binding and residual hybridization, modulating signal dynamics via tuneable electrostatic gating. These results underscore the promise of aptamer engineering and DNA nanotechnology for developing high‐performance biosensors with programmable behavior, offering new avenues for real‐time sensing, dynamic molecular recognition, and integrated diagnostic technologies.

## Graphical abstract

Aptamer‐based single‐walled carbon nanotube (SWCNT) field‐effect transistor (FET) biosensors are developed for label‐free, real‐time biomarker detection. Truncated DNA aptamers enhance sensitivity through improved conformational switching. A toehold‐mediated strand displacement (TMSD) strategy optimizes functionalization and simplifies fabrication. The addition of a protective strand introduces bistable signal outputs, highlighting the potential of DNA nanotechnology for high‐performance biosensors.

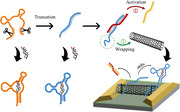

## 1 Introduction

Biomarkers play a critical role in early disease diagnosis and physiological condition monitoring [1]. Traditional analytical techniques such as liquid chromatography–mass spectrometry (LC‐MS) [2, 3] enzyme‐linked immunosorbent assays (ELISA), [4, 5] and chemiluminescence assays, [6, 7] offer high sensitivity and specificity but are often time‐consuming, labour‐intensive, and reliant on bulky instrumentation; this limits their utility for point‐of‐care applications. As a promising alternative, nanoscale biosensors based on field‐effect transistor (FET) architectures have garnered significant attention due to their compact design, label‐free readout, and real‐time detection capabilities [8, 9, 10, 11]. Their miniaturized format enhances portability, while the direct electrical signal transduction enables real‐time read‐out, as well as high sensitivity by exploiting the high surface‐to‐volume ratio and the close proximity between the recognition interface and the conductive channel [11, 12, 13].

Among the potential channel materials for biosensing FETs, single‐walled carbon nanotubes (SWCNTs) have emerged as promising candidates, offering high conductivity, nanoscale dimensions, and chemical tunability [14, 15, 16, 17, 18, 19, 20]. However, non‐specific physisorption of biomolecules onto SWCNT sidewalls can impair sensing specificity. To address this, molecular recognition elements such as organic molecules, [21] polymers, [22] antibodies, [23] proteins, [24, 25, 26] and DNA aptamers [16, 17, 19, 27, 28] have been immobilized on SWCNTs to improve sensing specificity [29]. Among these, DNA aptamers stand out due to their strong and specific binding affinity to target molecules through well‐defined secondary and tertiary structures, along with thermal and chemical stability, batch‐to‐batch consistency, and reusability [3, 29, 30, 31, 32, 33, 34, 35, 36].

Aptamers can be immobilized onto SWCNTs through either covalent or non‐covalent strategies, such as radical coupling reactions or π–π interactions by pyrene moieties [38, 39, 40]. Among various non‐covalent approaches, DNA wrapping has emerged as one of the most promising strategies for functionalizing SWCNTs, particularly for bio‐related applications [41, 42] as it enhances the dispersity of SWCNTs in aqueous environments without affecting their intrinsic electrical properties. Additionally, the programmable nature of the DNA sequence enables the control of its binding affinity to SWCNTs of different chirality [43, 44, 45], Moreover, DNA‐wrapped SWCNTs allow for the interfacing of functional moieties either through direct hybridization at a “sticky end” of the DNA wrapping sequence [46] or incorporating functional groups at the 3’ or 5’ end for covalent linkage [19]. These strategies offer solution processable routes for the fabrication of both optical and electrical SWCNT biosensors with aptamers, [19, 46, 47, 48, 49, 50, 51] including FET sensing devices [29].

The sensing mechanism and performance of SWCNT‐aptamer FET biosensors are predominantly governed by electrostatic gating induced by target‐induced conformational changes of the aptamer, [16, 17, 37] while the detectable range of this charge modulation is constrained by Debye screening in the electrolyte solution [8, 51, 52]. In this regard, it is of paramount importance to control the aptamer‐transducer interface toward the optimization of the FETs biosensing response. Different strategies have been employed toward this end, including interface optimisation with nanoscale control, [53] tuning Debye screening [54] and the functionalization strategy employed to tether the aptamer to the CNT transducer [16]. In this regard, aptamer engineering [55] has proven important, typically via chemical modification and truncation, [35] so to optimize aptamer's structural changes upon biomarker recognition and maximize the sensors’ response. Recent structure‐guided studies have further demonstrated that target functional group–informed aptamer engineering is critical for achieving high‐affinity and selective recognition of challenging small‐molecule targets [56].

In this study, we first tested in SWCNT‐FET biosensors, a recently devised truncated aptamer (CSS.3_cut3) with enhanced conformational switching for optimal cortisol binding [35]. We observed increased sensitivity in our sensing devices compared to ones built employing the original untruncated aptamer. Subsequently, we explored a dynamic functionalization approach for the in‐solution interfacing of the truncated optimized CSS.3_cut3 aptamer to SWCNTs, employed in FET biosensors. Specifically, we incorporated a Toehold‐Mediated Strand Displacement (TMSD) strategy [57, 58], aiming at streamlining the functionalization process and further optimizing the sensing performance. Initially developed for molecular logic and nanomachines, [59] TMSD enables controlled strand exchange via short, single‐stranded toehold regions, and has been widely adopted in synthetic biology, [60] nanomedicine, [61] and biosensing [62, 63, 64]. However, its integration into electronic sensing platforms, particularly FET‐based nanosensors, remains exceptionally rare [65]. Compared to our previously developed strategy based on the covalent attachment of the aptamer on the DNA‐wrapping the CNTs, [16, 19] the TMSD‐processed SWCNT‐FET biosensors fabricated in this study exhibited improved sensitivity toward cortisol detection, enabled by a simpler and more efficient functionalization process. Moreover, a partially mismatched DNA base‐pair was introduced into the TMSD design to ensure optimal activation of the DNA aptamer region after wrapping onto the SWCNT [66]. This design allowed the aptamer to retain its recognition capability even while hybridized with the protective strand, thereby enabling the study of competitive interactions between the target molecule (cortisol) and the protecting strand. Interestingly, this altered the typical stepwise current signal changes over time observed in our SWCNT‐FET sensors, to instead give rise to two distinct current states—a high (*G_high_*) and a low (*G_low_*) conductance level‐upon exposure to cortisol, suggesting a reversible association and dissociation of cortisol with the aptamer, mediated by competition with the partially complementary protection strand. Thus, the TMSD strategy not only enhances sensing performance but also provides new insights into tuneable electrostatic gating and surface‐bound conformational dynamics in aptamer‐SWCNT‐FET biosensors. Our findings highlight the potential of this design strategy for the development and optimization of biosensors capable of real‐time biomarker detection.

## 2 Results and Discussions

### 2.1 Optimizing Aptamer Structural‐Switching in SWCNT‐FET Biosensors Through Truncation

To investigate the impact of aptamer engineering on the workings of FET‐based biosensors, we tethered a cortisol‐binding aptamer CSS.3 and its truncated variant CSS.3_cut3 onto DNA‐wrapped SWCNTs via a click chemistry strategy we already developed and employed successfully in the fabrication of SWCNT‐based sensing devices [19] [Figure S1]. These hybrids were then assembled in FET device configuration on prepatterned electrodes via dielectrophoresis (DEP) as previously demonstrated [19]. The detailed procedure is provided in the SI.

The truncated variant CSS.3_cut3 was derived from the parent CSS.3 aptamer by removing 6 nucleotides from the 5’ end and 3 nucleotides from the 3’ end, while preserving the core target‐binding region [35]. Despite the truncation of the stem in the CSS.3 sequence weakening the cortisol binding affinity (Kd increased from 660 nm to 990 nM), this truncation reduces structural constraints present in CSS.3, promoting a more pronounced unstructured‐to‐structured transition upon cortisol binding. Such structural modification is intended to enhance electrostatic gating effects within the Debye length, thereby improving the sensitivity of SWCNT‐FET biosensors (full sequences provided in the Supporting Information).

**FIGURE 1:**
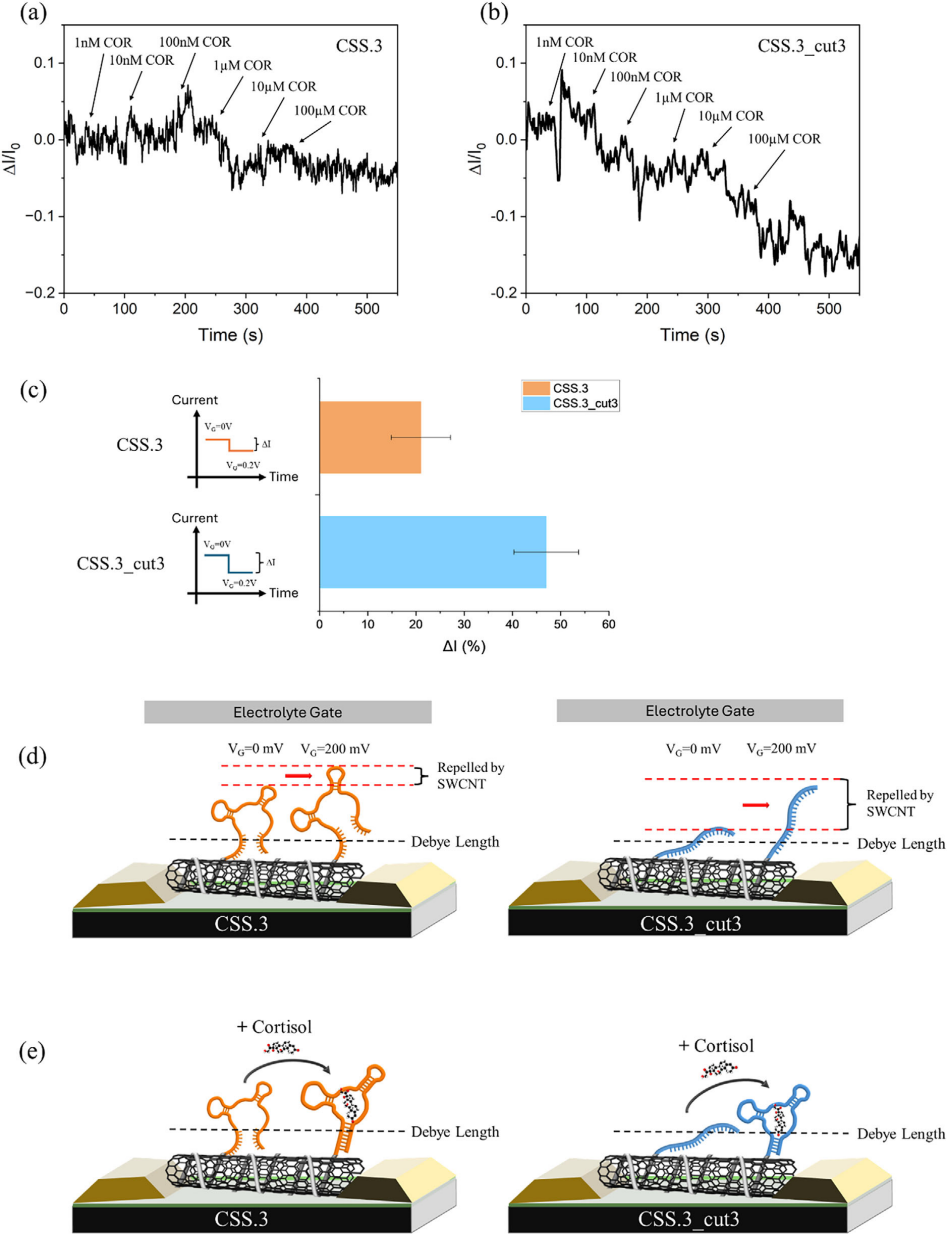
Representative real‐time cortisol detection responses of (a) CSS.3 and (b) CSS.3_cut3 SWCNT FET biosensors. (c) The device conductance changes ΔI of the CSS.3/CSS.3_cut3‐SWCNT devices at top electrolyte gate V_G_ = 0 and 200 mV (sample size n = 20); (d) Schematic illustration of the potential structural changes of the CSS.3 and CSS.3_cut3 aptamers on the SWCNT device with top electrolyte gating control; (e) Schematic illustration of the potential structural changes in the cortisol detection by the CSS.3 and CSS.3_cut SWCNT FET biosensors.

We tested the responses of the CSS.3/CSS.3_cut3 devices to cortisol. As shown in Figure 1a,b, the representative electrical response traces of both SWCNT‐FET biosensors exhibited a decreasing electrical response upon increasing cortisol concentration. However, the truncated variant CSS.3_cut3 on average gave rise to greater device responses under low cortisol concentration (especially at nanomolar levels), and higher sensitivity to varying biomarker levels introduced at distinct time intervals (Figure S2). This superior performance of the CSS_cut3 biosensor can be attributed to its unique structure and conformational‐switching behavior after aptamer engineering (truncation) [35]. Both CSS.3 and CSS.3_cut3 undergo conformation changes upon cortisol binding, but with different structural transitions. Unlike the parent CSS.3 aptamer, which possesses a defined secondary structure with constrained conformation freedom and undergoes a subtle structured‐to‐structured transformation, the truncated CSS.3_cut3 lacks a secondary structure in the unbound state and undergoes a marked unstructured‐to‐structured transformation.

To further validate the structural difference of the two aptamers on SWCNT devices and investigate the origin of the superior sensing performance obtained employing the truncated aptamer, we measured the device current (source‐drain I_SD_ at voltage V_SD_ = 100 mV) as a function of top electrolyte gate voltage. A positive gate induces negative charges on the CNTs [67] likely to repel the negatively charged aptamers from the surface, hence reducing hole density in the p‐type nanotube semiconductor, eventually decreasing I_SD_. However, this change of device conductance (ΔI) could be influenced by the motion behavior of the aptamers on the surface. Figure 1c shows the ΔI as a function of the top electrolyte gating (at 0 and 200 mV) for the CSS.3, and CSS.3_cut3 devices: the ΔI of the CSS.3_cut3 devices is two times the one measured for the CSS.3 devices. As illustrated in Figure 1d, this strongly indicates that the unstructured CSS‐cut3 aptamer is pushed further away from the CNTs surface by the applied positive top electrolyte gate, as expected due to its unstructured nature. The transfer curve dynamics (Figure S3) further support this interpretation. The CSS.3_cut3 device exhibits a larger hysteresis loop than the CSS.3 control, which in turn shows a nearly hysteresis‐free, reversible curve. We attribute these observations to the unstructured nature of the CSS.3_cut3 aptamer.

When CSS.3_cut3 binds to cortisol, it undergoes a significant ‘unstructured‐to‐structured’ transformation [35]. This structural change causes a larger portion of its negatively charged backbone to extend beyond the Debye length, inducing a more pronounced decrease in hole accumulation and a corresponding decrease in the measured I_DS_ (Figure 1a,b,e). Here, hole accumulation refers to the local concentration of charge carriers (holes) in the p‐type SWCNT channel. Cortisol binding triggers the conformational switching of the aptamer, thereby altering the local electrostatic environment within the Debye length. This change in electrostatics modulates the local hole concentration in the channel, resulting in a measurable change in device current. Therefore, the dynamic‐driven, large‐amplitude conformational shift in CSS.3_cut3 is the key contributor to its enhanced sensor response and higher sensitivity to cortisol. This highlights how the optimization of conformational‐switching in aptamers is key for enhancing the sensitivity of FET‐based biosensors.

### 2.2 Toehold‐Mediated Strand Displacement (TMSD) for Enhanced SWCNT‐FET Functionalization

**FIGURE 2:**
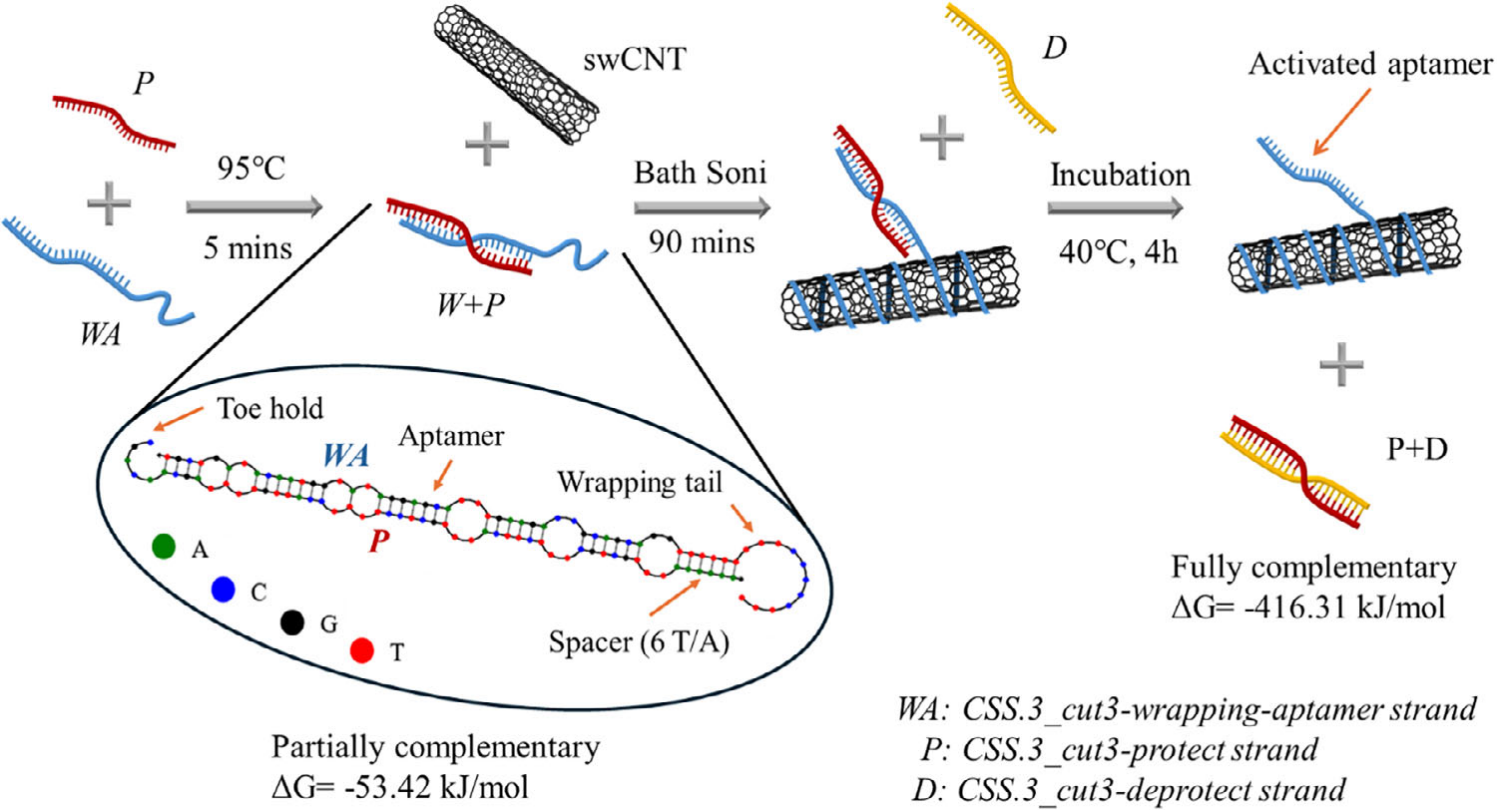
Schematic illustration of the DNA strand design and functionalization process of single‐walled carbon nanotubes (SWCNTs) using the toehold mediated strand displacement (TMSD). The CSS.3_cut3 wrapping‐aptamer strand (WA, blue) is designed to have the wrapping tail domain, spacer domain, CSS.3_cut3 aptamer domain, and toehold domain. Consequently, a CSS.3_cut3 protect strand (P, red) that is partially complementary with W‐A strand, and a CSS.3_cut3 deprotection strand (D, yellow) that is complementary with P strand.

In order to take advantage of the high sensitivity of the CSS.3_cut3 aptamer, we introduced an alternative TMSD functionalization method in the assembly of our aptamer‐CNT interfaces. The functionalization workflow and strand design are illustrated in Figure 2. This method relies on the hybridization reactions between three single‐stranded DNA components (ssDNAs): a wrapping‐aptamer strand (WA), a protection strand (P), and a deprotection strand (D). The WA strand serves as the primary functional strand, incorporating both the CSS.3_cut3 aptamer and a wrapping DNA tail, which enables attachment to the SWCNT surface while preserving the binding affinity to the target analyte [48]. To maintain optimal spacing between the aptamer and SWCNT, a six‐thymine (T_6_) spacer was introduced.

Prior to attachment of the WA strand onto the SWCNT surface, the P strand was hybridized with the aptamer region of WA. This step was designed to shield both the aptamer and the spacer region from nonspecific adsorption with the nanotube surface, ensuring that only the intended wrapping tail of WA engaged with the SWCNT. We deliberately designed the WA‐P duplex to be only partially complementary (Gibbs free energy = –53.42 kJ/mol, computationally estimated; see the SI and Figure S4), while the P‐D duplex was fully complementary and significantly more stable (Gibbs free energy = –416.31 kJ/mol, see the SI and Figure S4). This mismatch‐elimination strategy introduces a hidden thermodynamic drive, as described by Haley et al. [66], which strongly favours forward strand displacement while minimally affecting the kinetics. Building on this design, we incorporated a 5‐nucleotide toehold on the P strand. This length was chosen based on prior studies showing its effect on strand exchange efficiency [58] and its successful application on SWCNTs [68]. The toehold and mismatch setup enabled rapid and directional strand displacement, leading to efficient release of the P strand from the WA–SWCNT nanohybrid upon addition of the D strand. This asymmetric thermodynamic and kinetic design was critical for our assembly strategy, as it ensured ‐at command—complete displacement of the P strand and full exposure of the WA strand's aptamer region, which is essential for subsequent cortisol detection.

**FIGURE 3:**
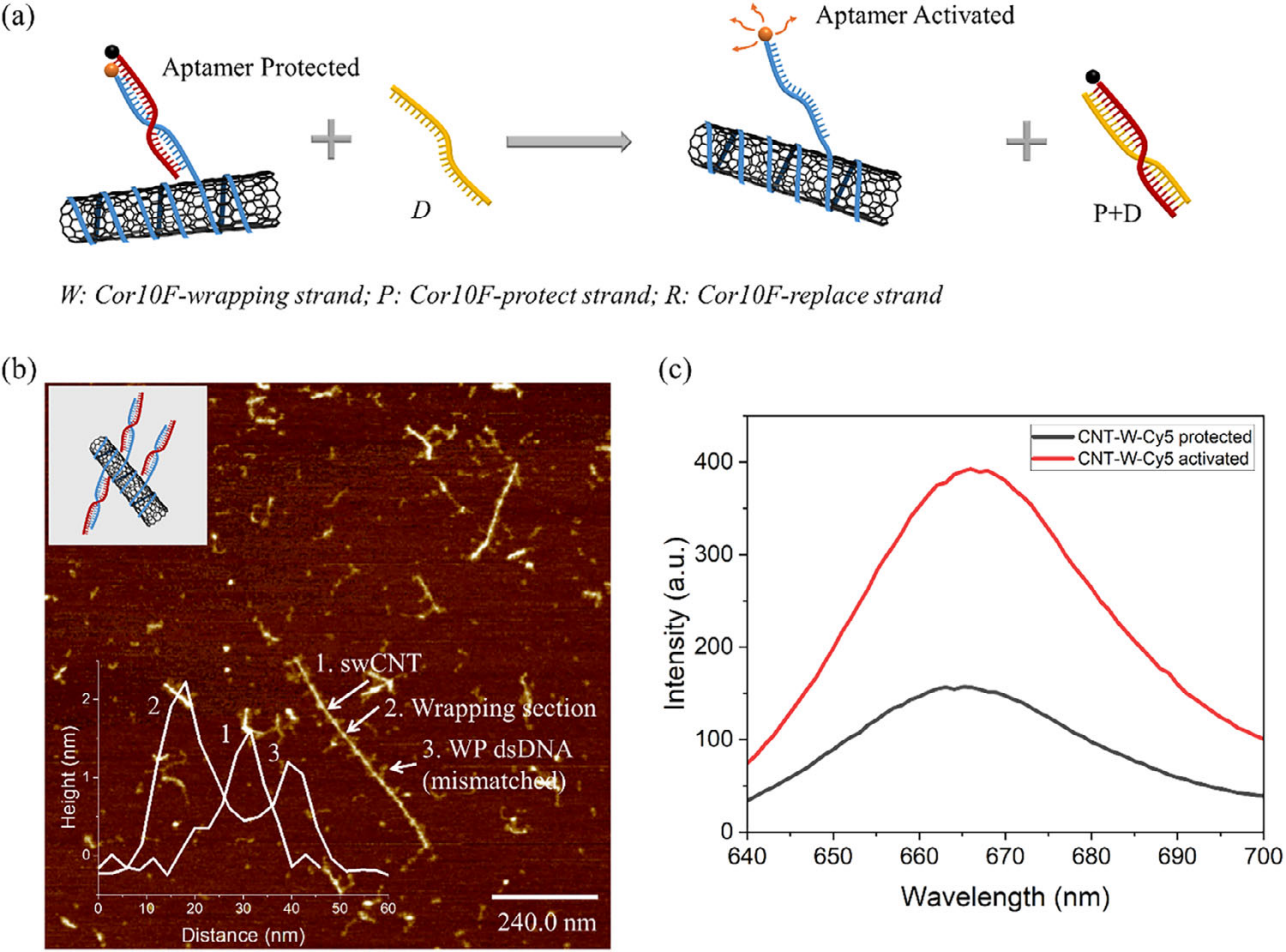
(a) Schematic illustration of the fluorophore‐labelled TMSD process; (b) topographical AFM images of the P strand‐protected WA‐SWCNT nanohybrid; (c) fluorescence spectroscopy of the CSS.3_cut3 wrapped SWCNT before and after protection.

Aptamer attachment to the SWCNT surface was corroborated by atomic force microscopy (AFM). As shown in Figure 3b, topographical imaging of WA‐P SWCNT hybrids deposited on mica revealed protruding features consistent with partially mismatched WA‐P duplexes extending from the nanotube surface. Raman spectroscopy investigations provided further evidence of the assembly of SWCNT with WA. Figure S5 displays the G^+^ and G^−^ bands at 1575.1 and 1540.9 cm^−^^1^, respectively, characteristic of the Raman spectra of pristine SWCNTs. The G^+^ band, typically observed near 1580 cm^−^^1^, arises from longitudinal vibrations of carbon atoms along the nanotube axis, while the G^−^ band corresponds to circumferential vibrations [69, 70]. Following attachment of the WA strand before and after activation (Figure S5, red and blue curves), the G^−^ peak was significantly suppressed, likely attributable to the inhibition of circumferential vibrations due to the tight helical wrapping of the DNA wrapping tail around the nanotube surface [69]. Additionally, the G‐band became narrower and exhibited an upshift relative to pristine (7,6) SWCNTs, indicating a charge‐transfer interaction between the WA strand and the SWCNT [69, 71, 72, 73]. These spectral changes are an indication of the successful and stable functionalization of WA strands on the SWCNTs, which in turn remain unaffected by the subsequent TMSD‐driven activation of the aptamer. Additionally, native gel electrophoresis confirmed the displacement reaction: see Figure S6a.

To unambiguously validate our TMSD methodology, we employed fluorescence spectroscopy. The WA and P strands were labelled with a Cy5 fluorophore and a black hole quencher (BHQ), respectively. As illustrated in Figure 3a, the hybridization of WA and P brings Cy5 and BHQ into proximity, resulting in fluorescence quenching (Figure 3c, black curve; the protected aptamer is not completely quenched likely due to suboptimal spectral overlap and/or spatial arrangement between the fluorophores). Upon addition of D, strand displacement occurs, releasing WA from P and separating the fluorophore from the quencher, thereby restoring fluorescence (Figure 3c, red curve).

### 2.3 Integration of Toehold‐Mediated Strand Displacement (TMSD) in SWCNT‐FET Biosensing

TMSD has been widely applied in biosensing due to its enzyme‐free, isothermal, and programmable nature, enabling high specificity and signal amplification for diverse targets [62]. While conventional TMSD‐based biosensors primarily rely on fluorescence labelling for signal output, [60, 62] optical methods often require external labelling, complex instrumentation, and are not ideally suited for real‐time or point‐of‐care sensing applications. Integrating TMSD with SWCNT‐FET biosensors can offer a powerful alternative, enabling direct electrical readout of molecular recognition events.

**FIGURE 4:**
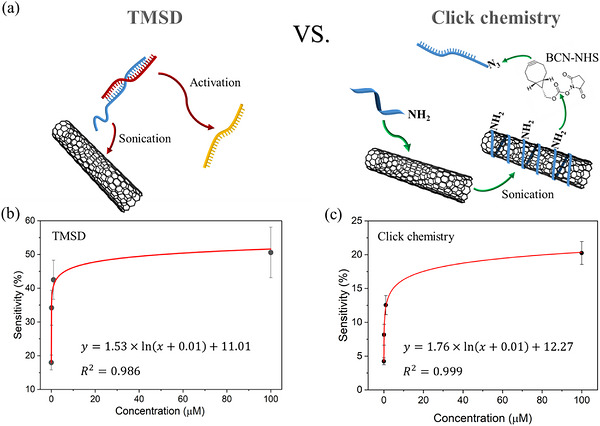
(a) Schematic illustration comparing the click chemistry and TMSD functionalization methods. Calibration curves of real‐time cortisol detection via (b) TMSD and (c) click chemistry functionalized CSS.3_cut3 SWCNT FET biosensors (sample size = 10 devices for each methodology).

Following the D strand–mediated deprotection in our TMSD strategy (see again Figure 3a), the activated CSS.3_cut3–SWCNT nanohybrid was assembled into a FET device using DEP, as (Figure S7). As shown in Figure 4a, the TMSD‐based approach is a more facile strategy via direct wrapping of the aptamer onto pristine SWCNTs. It simplifies the fabrication process minimizing time‐consuming reaction and purification steps, toward the optimization of reaction efficiency, reduced oligonucleotide modification costs, and full solution processability in the fabrication of SWCNT‐FET biosensors.

A representative trace of real‐time cortisol detection using the TMSD‐functionalized SWCNT‐FET device is shown in Figure S8: the source‐drain current exhibited stepwise decreases in response to increasing cortisol concentrations, consistent with binding‐induced gating effects [19]. In the cortisol binding event, the aptamer undergoes conformational changes that reposition its negatively charged phosphate backbone relative to the SWCNT channel. This shift reduces the local hole concentration in the p‐type SWCNT transducer within the Debye length, resulting in a decrease in conductance through electrostatic gating effects, as we and others have demonstrated [16, 17, 19, 26, 74]. Notably, TMSD‐functionalized biosensors exhibited overall significantly enhanced sensitivity (∼50%) compared to click chemistry–functionalized counterparts (∼20%); notably at low concentration (1 nM), we observed a sensitivity enhancement of ∼15% for TMSD vs. 4% for click‐chemistry, as shown in Figure 4b,c. This level of sensitivity is sufficient for clinical applications, particularly in salivary diagnostics where cortisol concentrations are typically low [75]. The sensors can be restored after exposure to high‐concentration urea, which dissociates the aptamer–cortisol complex, highlighting the system's reusability. Stability testing showed consistent sensor performance over multiple detection cycles and after one month of storage (Figure S9).

### 2.4 Exploring the Impact of Aptamer Configurations on Sensor Behavior in TMSD‐FET Biosensors

**FIGURE 5:**
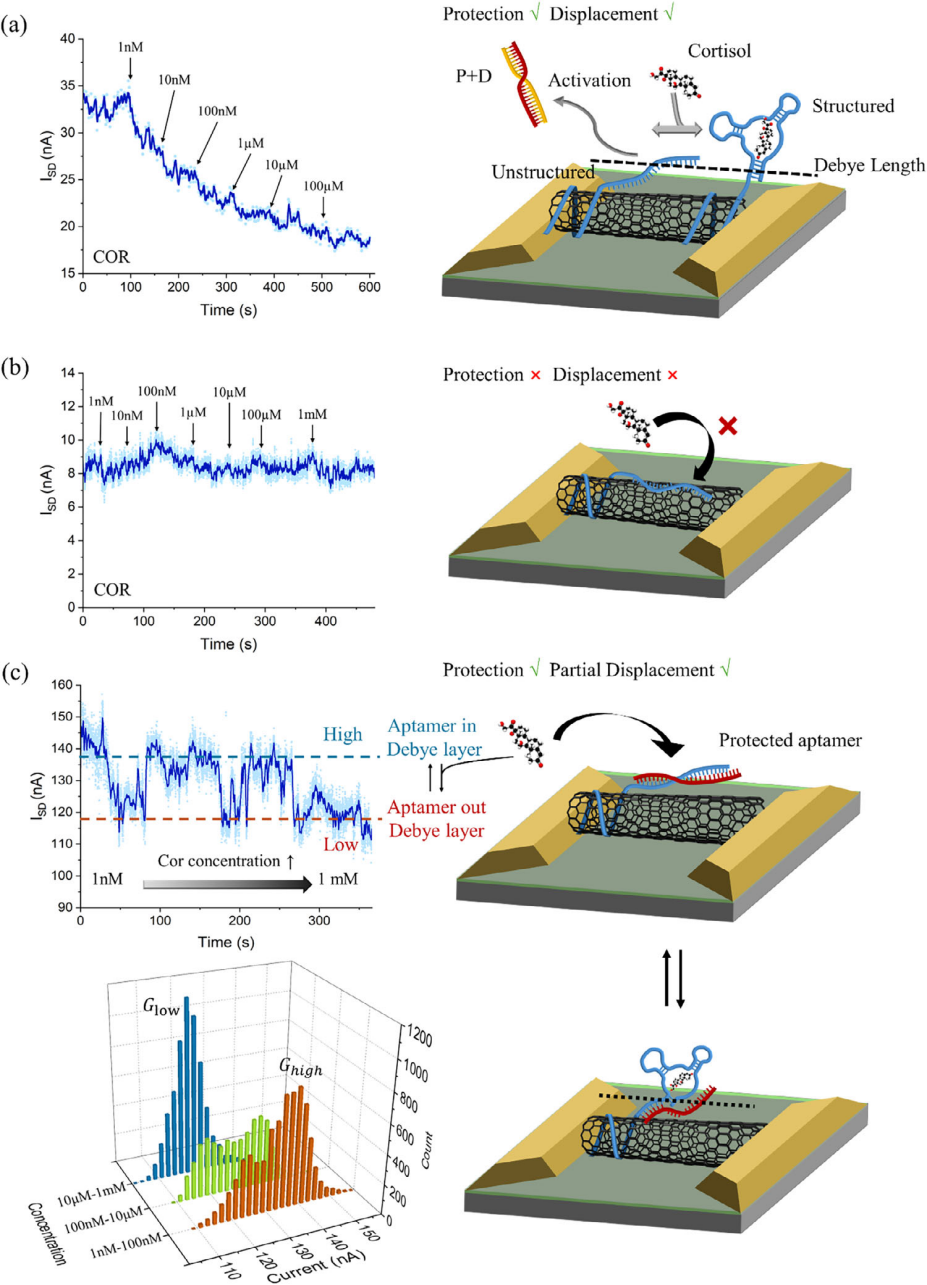
Real‐time current responses and mechanistic model hypothesis of SWCNT‐FET biosensors employing toehold‐mediated strand displacement (TMSD) for cortisol sensing.(a) Device undergoes full TMSD‐process, with P strand protection and D strand displacement activation. The sensor exhibits a stepwise decrease in conductance with increasing cortisol concentrations (1 nM to 1 mM), consistent with aptamer conformational change and strand displacement within the Debye length. (b) Control device without TMSD‐process, W‐aptamer directly wrapped on SWCNT. The sensor shows neglectable response across the same concentration range, indicating the importance of protection and displacement steps.(c) Device undergoes partial TMSD‐process, with aptamer protection by P strand but leaves the partially complimentary P strand on the aptamer during cortisol detection. The sensor with partial mismatch designed P strand displays bistable current fluctuations upon cortisol addition (*G_high_* and *G_low_*), reflecting aptamer conformations alternately positioned inside and outside the Debye screening layer. Histogram analysis reveals the cortisol concentration dependence of the occurrence of the two distinct conductance states *G_high_* and *G_low_*. From low (1‐100 nM, the orange distribution), medium (100nM‐10 µM, the green distribution), to high (10µM‐1 mM, the blue distribution) cortisol concentration ranges, the device responds gradually transitioning from a *G_high_* to a *G_low_* dominant response.

Encouraged by the improved sensor performance, we next investigated how different aptamer configurations within each design element of the TMSD strategy affect device response and sensing behavior. Figure 5a shows the typical real‐time current response we observe for cortisol detection when using the TMSD‐functionalized SWCNT‐FET device. The TMSD methodology for SWCNT‐biosensor fabrication involves two key steps: the “protection” of the aptamer sequence during SWCNT functionalization and the “activation” of the aptamer by strand displacement (see Figure 2). To explore the contribution of each of these design elements, we first directly wrapped the W strands on the SWCNT without the P strand and demonstrated the necessity of the protection step. As shown in Figure 5b, the device shows a negligible cortisol response, indicating the aptamer is in a stable but inactive conformation. This observation can be rationalized through the inherently strong π–π stacking interactions between single‐stranded DNA and SWCNT surfaces, which significantly lower the Gibbs free energy, stabilizing the aptamer in a conformation inaccessible to cortisol.

We then re‐introduced the P strand on the complementary section of the WA strand wrapped around the SWCNT, but skipped the strand displacement step, leaving the aptamer in the protected form. The hybridized P strand can effectively desorb the aptamer (partially unwrapping the WA strand) from the SWCNT surface, driven by electrostatic interactions and spatial confinement effects, [76, 77] resulting in the device current level shift from a few nanoamps (Figure 5b) to hundreds of nanoamps (Figure 5c). This can be attributed to the enhanced negative charge density brought by the double‐stranded DNA structure within the Debye length, which electrostatically gates the SWCNT channel and facilitates hole transport, thereby boosting conductance [78].

Interestingly, if we add cortisol to perform the real‐time cortisol sensing experiment on the WA‐P‐protected SWCNTs (Figure 5c; Figure S10), the devices exhibit cortisol‐detection capabilities. Notably, the system switches to a distinct signalling behavior that closely resembles single‐molecule kinetics on aptamer‐functionalized SWCNT‐FET sensors, as previously reported [17]. In Lee et al.’s work, [17] a two‐level signalling mode originates from the adsorption and desorption of the aptamer backbone on the SWCNT surface via π‐π interactions, upon the addition of the target analyte, triggering the electrostatic gating. Differently, in our system, the P strand does not completely dehybridize from WA in the presence of cortisol, as confirmed by the lack of separation for the P band in gel electrophoresis experiments (Figure S6b). Therefore, dsDNA nature of the P‐protected aptamer –that is only partially hybridized to the aptamer in the WA‐P complex– likely promoted dynamic binding‐induced conformational changes in the aptamer upon cortisol recognition, giving rise to the two‐levels biosensing we observe in these electrical biosensors.

Specifically, we can decipher two signalling modes: a high conductance (*G_high_*) and a low conductance (*G_low_*), corresponding to the cortisol‐aptamer unbound and bound states. The aptamer section of the WA strand in the starting WA‐P hybrid (*G_high_*), likely undergoes a conformational rearrangement upon binding to the target analyte. This disrupts the partially mismatched WA‐P duplex hybridization, shifting the nucleotides' electrostatic charge further away from the SWCNT surface, i.e., a greater proportion of it will fall outside of the Debye length, with a consequential reduction in conductance to a low‐conductance state (*G_low_*). The reversible single molecule‐like signal behavior we observe, i.e., the switching from *G_high_* to *G_low_*, likely originates from the dynamic competition between aptamer‐cortisol binding and the aptamer's hybridization to the P strand (in the WA‐P hybrid). The degree of this conductance state transition is further dependent on cortisol concentration. As revealed in the histogram of the conductance states distribution in Figure 5c, the *G_high_* is dominant at the low cortisol concentration (1‐100 nM, orange state distribution); as the cortisol increases to a medium concentration range (100nM‐10 µM, green state distribution), the aptamers interact more with the cortisol causing a greater transition from *G_high_* to *G_low_*. At a high cortisol concentration level (10µM‐1 mM, blue state distribution), the binding interaction by the aptamer becomes dominant, causing the device response to saturate and falling to *G_low_*.

To understand the thermodynamic basis of signal generation in our sensor, we compared the Gibbs free energy (ΔG) of cortisol binding to the CSS.3_cut3 aptamer (–34.25 kJ/mol, as previously reported [35]) with the hybridization energy of the WA‐P duplex (–53.4 kJ/mol, estimated via a commercially available oligoanalyzer software; see the SI). As already mentioned, it is reasonable to attribute the observed dynamic current behavior in the SWCNT‐FET biosensor to a competitive binding equilibrium between the aptamer's recognition of cortisol and its partial hybridization to the P strand. A thermodynamic drive likely underlies the emergence of the reversible, bi‐level signal states (*G_high_* and *G_low_*), in contrast to the stepwise signal decrease typically observed in conventional irreversible TMSD‐based FET biosensors. By leveraging a programmable hybridization‐based interaction, this design introduces a distinct and controllable route to reversible signal transduction. While further studies are needed to quantitatively resolve the underlying kinetics, our findings suggest that such duplex‐mediated modulation of aptamer accessibility could serve as a general strategy for engineering dynamic and tuneable sensing behavior in FET biosensors.

## 3 Conclusion

In summary, we investigated the impact of aptamer engineering and optimal functionalization strategies in the performance of aptamer‐SWCNT FET‐based biosensors. Specifically, we tested a recently devised truncated aptamer (CSS.3_cut3) with optimal conformational switching for cortisol binding [35] and demonstrated the increased sensitivity of the corresponding FET biosensors (compared to employing the original untruncated aptamer). We then presented a DNA‐based strategy via TMSD for the interfacing of the aforementioned CSS.3_cut3 to SWCNTs; this resulted in a further enhancement in the sensitivity of FET biosensors. Incorporating a TMSD‐based functionalization scheme not only improved signal response but simplified the fabrication strategy by eliminating the need for multi‐step chemical coupling. Notably, devices incorporating a partially complementary protection strand to the aptamer in the TMSD SWCNT hybrids exhibited two distinct signalling modes: a bistable, two‐level behavior, in contrast to the gradual response of conventional deprotected sensors. This behaviour likely arises from the interplay between aptamer–target binding and residual strand hybridization, providing enhanced control over signal dynamics and more precise tuning of sensor response. We believe these findings establish TMSD as a versatile design element of general applicability for both sensor fabrication and signal control, offering potential new opportunities for real‐time biosensing, dynamic molecular detection, and integrated diagnostic platforms.

## 4 Experimental Section/Methods

### 4.1 Materials

Dulbecco's phosphate‐buffered saline (DPBS) was obtained from Thermo Scientific. The Au/Ti electrodes patterned Si/SiO_2_ chips were purchased from Conscience. (1R,8S,9s)‐Bicyclo [6.1.0] non‐4‐yn‐9‐ylmethyl N‐succinimidyl carbonate (BCN‐NHS), (7,6) enriched SWCNT, and all the other chemicals were purchased from Sigma–Aldrich. Oligonucleotides (single‐strand DNA, ssDNA) were purchased from IDT. The sequences are:

Wrapping sequence: 5’‐Amine‐TTTCCCCCTTT‐3’

CSS.3: 5’‐/AzideN/CTCTCGGGACGACGCCAGAAGTTTACGAGGATATGGTAACATAGTCGTCCC‐3’

CSS.3_cut3: 5’‐/AzideN/GGACGACGCCAGAAGTTTACGAGGATATGGTAACATAGTCGT‐3’

CSS.3_cut3‐wrapping sequence (W): 5’‐TTTCCCCCTTTTTTTTTGGACGACGCCAGAAGTTTACGAGGATATGGTAACATAGTCGT‐3’

CSS.3_cut3‐protect sequence (P): 5’‐CGACAACGATTTTGTTACCTTTTCCTCGTTTACTTCTTTCGTCGTTTAAAAAA‐3’

CSS.3_cut3‐deprotect sequence (D): 5’‐TTTTTTAAACGACGAAAGAAGTAAACGAGGAAAAGGTAACAAAATCGTTGTCG‐3’

CSS.3_cut3‐wrapping sequence (W) with fluorophore: 5’‐TTTCCCCCTTTTTTTTTGGACGACGCCAGAAGTTTACGAGGATATGGTAACATAGTCGT /3C5Sp/ ‐3’

CSS.3_cut3‐protect sequence (P) with quencher: 5’‐ /5IABkFQ/CGACAACGATTTTGTTACCTTTTCCTCGTTTACTTCTTTCGTCGTTTAAAAAA‐3’

### 4.2 Functionalization of Aptamer‐SWCNT Nanohybrid by Click Chemistry

1 mg (7,6) enriched SWCNT (Sigma–Aldrich) and 2 mg Amine‐DNA (IDT) were mixed with 1 mL 0.1 M NaCl solution. The resulting suspension was bath‐sonicated for 90 mins and then centrifuged for 30 min at 15000 rpm to remove the unwrapped SWCNT. As the DNA‐wrapped SWCNT has good dispersibility in water, the supernatant was kept as the final sample.

7 µL BCN‐NHS solution in DMSO (0.25 mg/14 µL) was mixed with 37.5 µL of 0.1 M sodium tetrahydrate solution (PH 8.5) and 5.5 µL MQ water to prepare the final BCN‐NHS solution. 50 µL prepared BNC‐NHS solution was added to amine‐terminated DNA‐wrapped SWCNT solution (10 times diluted, 1:1 equivalent) and left for incubation 2∼3 h at 25°C. The resulting solution was dialyzed for 1 h. After that, 5 µL azide‐aptamer and 5 µL DPBS buffer solution (10×) were added to 40 µL MQ water and then mixed with the prepared 100 uL BCN‐DNA‐wrapped CNT solution and incubated overnight. The aptamer will be tethered on the DNA‐wrapped SWCNT by click chemistry between the azide and BCN group.

### 4.3 Functionalization of Aptamer‐SWCNT Nanohybrid Toehold Mediated Strand Displacement (TMSD)

200 µL 300 µM CSS.3_cut3‐Wrapping‐aptamer strand (CSS.3_cut3‐WA), 200 µL 300 uM CSS.3_cut3‐Protection strand (CSS.3_cut3‐P), and 13.8 uL 3 M NaCl were mixed and heated to 95°C for 5 mins and then cooled down to room temperature for better conjugation. Then, 0.2 mg (7,6) SWCNT were dispersed in the CSS.3_cut3‐WA‐P solution by 90 min bath sonication (Branson 2500). The resulting suspension was centrifuged at 15060 rpm for 30 mins to remove the undispersed residue. After 1 h of dialysis (membrane), 30 µL 1000 µM CSS.3_cut3‐Replace strand was added to 100 µL CSS.3_cut3‐WA‐P‐SWCNT sample and incubate for 4 h. The resulting mixture was dialysed again to remove the excess of CSS.3_cut3‐D and the replaced CSS.3_cut3‐P‐D dsDNA. Herein, the aptamer was designed to have a wrapping tail that can directly bind with (7,6) SWCNT.

### 4.4 Fabrication of SWCNT FET Devices

The Au/Ti electrodes patterned chips purchased from Conscience contain 16 electrode pairs with a 300 nm gap between each pair. The nanoelectrode pattern was lithographed on a p‐doped Si/SiO_2_ wafer (300 nm SiO_2_ on Si) using laser and electron beam lithography. A thin Ti layer (5 nm) and a thick Au layer (45 nm) were then deposited onto the pattern via metal evaporation. The chips were cleaned by sonication in acetone and IPA for 5 min each, followed by 5 min of plasma cleaning. The DNA‐wrapped SWCNT was immobilized between Au electrode pairs on a SiO_2_/Si chip with a conventional field‐effect transistor structure by DEP. 5 µL droplet of 10‐times diluted aptamer‐SWCNT nanohybrid suspension was placed on the chip. A voltage bias of Vp‐p = 3 V and f = 400 kHz was applied across the source and drain electrodes for 2 min. The chip was then washed with Milli‐Q water and dried under N_2_.

### 4.5 Electrical Measurement

Electrical measurement was performed on a probe station (PS‐100, Lakeshore) connected with a Keithley 4200 SCS semiconducting parameter analyser. 5 µL sensing buffer (50 mM Tris‐HCl, 100 mM NaCl, and 5 mM MgCl_2_, pH = 7.4) was added to cover the devices and incubate 2 mins for stabilization. A 0.1 V bias was then applied, and the source‐drain current (I_SD_) was recorded upon the addition of cortisol solution from low (nM) to high (µM) concentration, with a 60s interval between successive additions. Subsequently, a drop of 8 M urea solution was cast on the device to clean the captured cortisol, making the device reusable. Finally, the chip was rinsed with MilliQ water and dried with N_2_. The cortisol solutions with a concentration from low to high were prepared by cascade diluting the 10 mM cortisol solution (in methanol) with the sensing buffer (50 mM Tris‐HCl, 100 mM NaCl, 5 mM MgCl_2_, pH 7.4).

## Funding

U.S. Air Force Office of Scientific Research under award FA8655‐21‐1‐7003. This work was supported by the National Research Foundation of Korea (NRF) grant funded by the Korea government (MSIT)(RS‐2025‐00523684). This work was supported by the Institute of Information & Communications Technology Planning & Evaluation (IITP) grant funded by the Korea government (MSIT) (RS‐2025‐02304986).

## Supporting information




**Supporting File**: advs75328‐sup‐0001‐SuppMat.pdf.

## Data Availability

The data that supports the findings of this study are available in the supplementary material of this article.
