# Supplementary material for: Optimization of SWCNT‐FET Biosensors by Aptamer Engineering and Toehold‐Mediated Strand Displacement
Source: Adv Sci (Weinh). 2026 Apr 16;13(38):e75328. doi: 10.1002/advs.75328 (PMC13335490; doi:10.1002/advs.75328)
Supplement: Supplementary file 1 — Supporting File: advs75328‐sup‐0001‐SuppMat.pdf. [file ADVS-13-e75328-s001.pdf]

Supporting Information

Optimization of SWCNT-FET Biosensors by aptamer engineering and Toehold-Mediated Strand Displacement

Haosen Miao, Tingting Zheng, Houlin Yu, Griiriraj Manoharan, Gustavo Sant'Anna, Nicholas Bedfrod, Jorge L. Chávez, Chang-Seuk Lee, and Matteo Palma

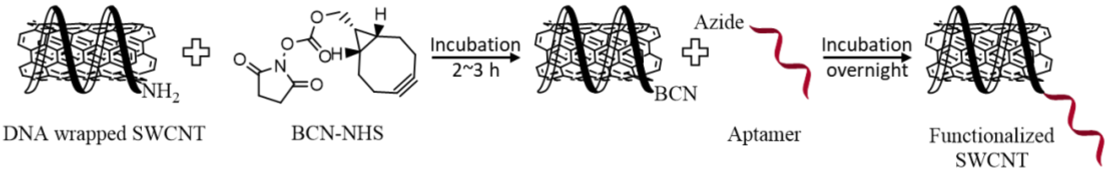

Figure S1. Schematic illustration of the click chemistry functionalization method

1. Calibration curve of real-time detection

Based on real-time detection data, calibration curves were constructed for cortisol. During the experiment, sequential additions of varying analyte concentrations led to stepwise decreases in current, with each addition reaching a distinct plateau. The initial baseline current (I_0) was determined by averaging the current prior to analyte addition. For each analyte concentration, the corresponding steady-state current (I) was calculated by averaging the current over the plateau region. These values were used to determine the sensor's sensitivity (S) to each analyte concentration, as described in Equation (1).

$$S = \frac{|I-I_0|}{I_0} \times 100\% \qquad (1)$$

The data was plotted and fitted by using equation (2)

$$y = a \times \ln(x-b) + c$$

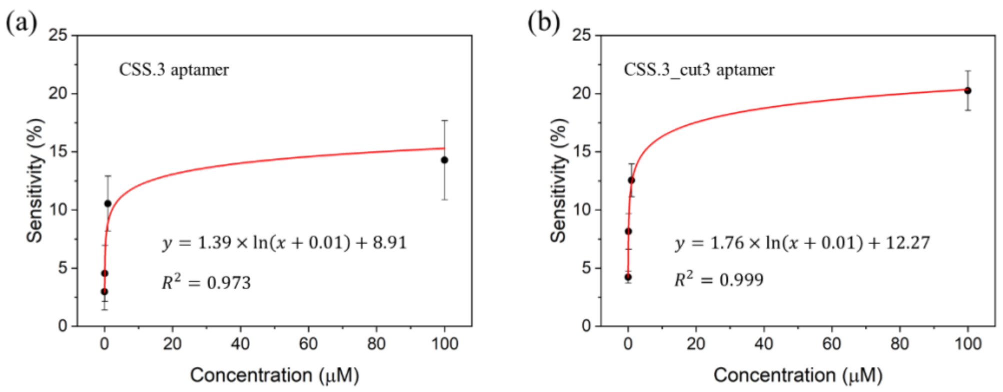

Figure S2. Calibration curves of the devices functionalized with (a) CSS.3 aptamer and (b) CSS.3_cut3 aptamer (sample size = 10 devices for each).

2. Top electrolyte gate voltage sweep

Dual voltage sweeps from -200mV to +200mV were applied via the top electrolyte gate. The transfer curves in Figure S3 show distinct hysteresis behaviour, while the truncated CSS.3_cut3 devices exhibit more pronounced hysteresis than the parent CSS.3.

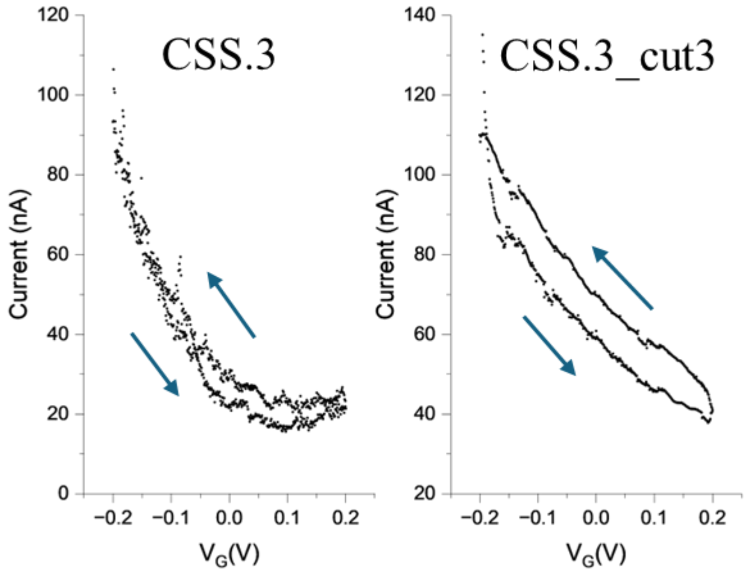

Figure S3. Duel top electrolyte gate sweep on CSS.3/CSS.3_cut3 devices

3. Estimation of Gibbs Free Energy for Aptamer–Target and DNA Hybridization Interactions

To gain thermodynamic insight into the sensing mechanism, we calculated the Gibbs free energy (ΔG) for both cortisol–aptamer binding and aptamer–protection strand (WA–P) hybridization. The ΔG of cortisol binding to the CSS.3_cut3 aptamer was estimated using the standard thermodynamic relationship:

$$\Delta G = -RTlnK_A$$

where R is the universal gas constant (1.987×10^{-3} kcal·mol^{-1}·K^{-1}), T is the temperature in Kelvin (298.15 K), and K_A is the association constant (reciprocal of the reported dissociation constant K_D):

$$K_A = \frac{1}{K_D}$$

The dissociation constant K_D for cortisol–CSS.3_cut3 binding was obtained from literature reports.[35] For the WA–P duplex, the hybridization free energy was computed using the IDT OligoAnalyzer™ Tool[2]. These values allowed comparison of the relative stability between aptamer–target binding and DNA hybridization, providing insight into the equilibrium dynamics underlying strand displacement and sensor activation.

The Gibbs free energy (ΔG) for WA-P and P-D duplex were estimated by IDT OligoAnalyzer™ Tool[2]:

```
Delta G: -12.77 kcal/mol  Base Pairs: 7    WA-P duplex
5' TTTCCCCCTTTTTTTTTGGACGACGCCAGAAGTTTACGAGGATATGGTAACATAGTCGT            WA
             ::::::  ::::::  ::::::  |||||||  :  :::::::  :  ::::
3'           AAAAAATTTGCTGCTTTCTTCATTTGCTCCTTTTCCATTGTTTTAGCAACAGC   P

Delta G: -99.5 kcal/mol  Base Pairs: 53    P-D duplex
5' TTTTTTAAACGACGAAAGAAGTAAACGAGGAAAAGGTAACAAAATCGTTGTCG              D
   |||||||||||||||||||||||||||||||||||||||||||||||||||||
3' AAAAAATTTGCTGCTTTCTTCATTTGCTCCTTTTCCATTGTTTTAGCAACAGC              P
```

Figure S4. The computational estimated Gibbs free energy of the WA-P and P-D duplexes in TMSD process via IDT OligoAnalyzer™ Tool[2]

4. Raman

RENISHAW inVia™ Raman microscope was used to measure the Raman spectrum of the SWCNT with a 442 nm laser source. The obtained Raman peaks were fitted in Origin@.

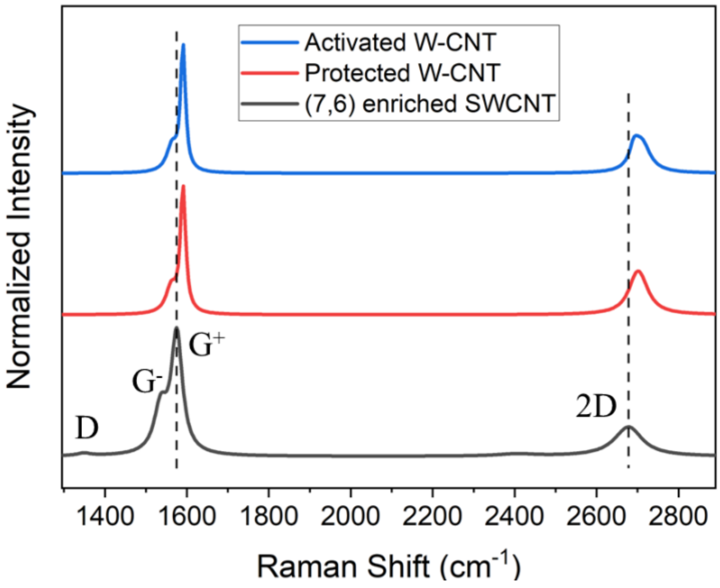

Figure S5. Raman spectroscopy of the TMSD functionalized SWCNT.

5. Gel electrophoresis

The 12% Native-PAGE gel electrophoresis was used to characterize the strand displacement. 1.2 mL 10× TBE, 0.8 mL H_2O, and 4 mL 30% Acrylamide bisacrylamide 19:1 was mixed together. Then, 25 µL 30% APS and 5 µL TEMED were added to the solution with gentle shaking to remove the bubbles. The prepared gel was quickly added to the gel plate before it was cured. The prepared gel plate was set up in the electrophoresis kit filled with 0.5 × TBE running buffer. WA, P, D, WA-P, P-D, SWCNT-WA-P, and SWCNT-WA-(P-D) solutions were diluted to 20 µM. After that, 10 µL of each solution was mixed with 2 µL 6× loading buffer and added to the prepared gel plate. 100 V voltage was applied for 3 hours to complete gel electrophoresis. Lanes containing individual strands (WA, P, D) and binary combinations (WA-P, P-D) served as controls. In the SWCNT–WA-P-D sample lane, distinct bands corresponding to the displaced P-D duplex and excess D strand were observed, confirming successful strand displacement.

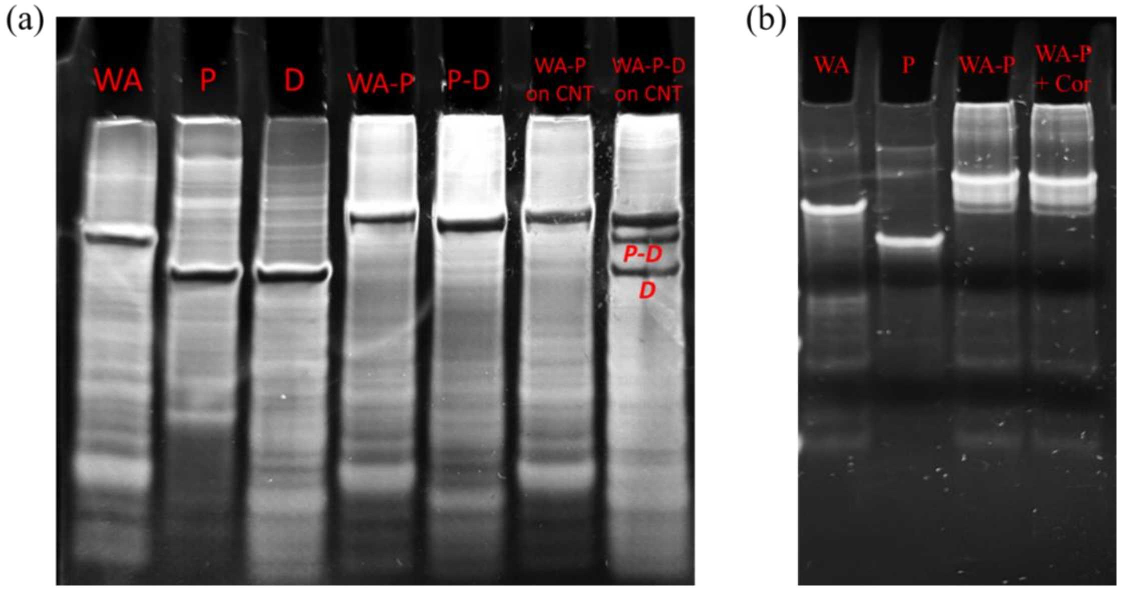

Figure S6. Gel electrophoresis of (a) the TMSD-processed aptamer-SWCNT nanohybrid; (b) the WA-P hybrid with cortisol addition

6. AFM imaging

The imaging was performed with a Bruker Dimension Icon Atomic force microscope (AFM) in Peak Force Quantitative Nanomechanical Mapping (QNM) mode using aluminium-coated probes (Scan analysis Air; Bruker). The SWCNT-aptamer solutions were diluted in TAE, 12.5 mM $MgCl_2$ buffer solution, and then deposited onto freshly cleaved mica for 10 mins incubation. Subsequently, the sample was blow-dried by nitrogen gas and then washed with 100 µL MiliQ water to remove the excess salt residues. The resulting topographic images were analyzed by Nanoscope Analysis 1.7 software.

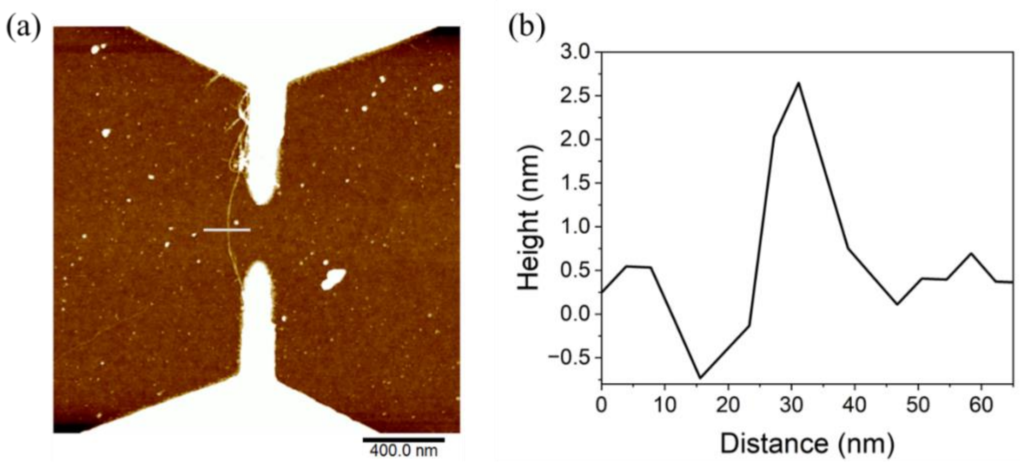

Figure S7. (a) Topological AFM image of the dielectrophoresis-immobilized SWCNT FET functionalized by TMSD. (b) The height profile of the immobilized SWCNT channel.

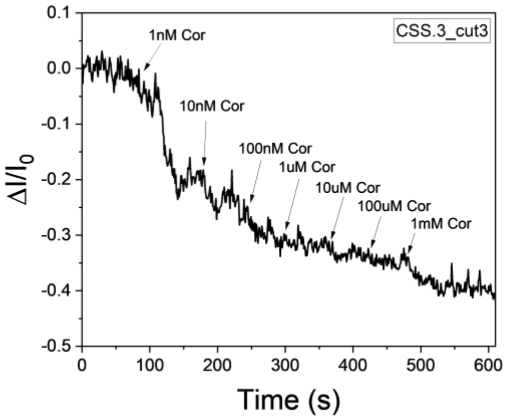

Figure S8. Representative real-time cortisol detection responses of the TMSD functionalized CSS.3_cut3 SWCNT FET biosensors.

7. Stability test

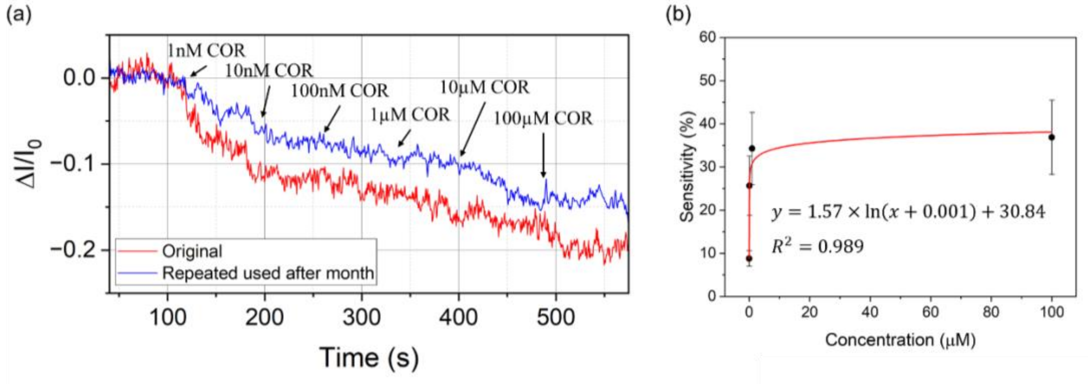

Figure S9. (a) Real-time cortisol detection of the TMSD functionalized CSS_cut3 SWCNT biosensor before (red curve) and after (black curve) repeated uses after a month. (b) Calibration curves of the devices that repeated used after a month

8. Real-time device response of the SWCNT-FET device with P-protected aptamer region

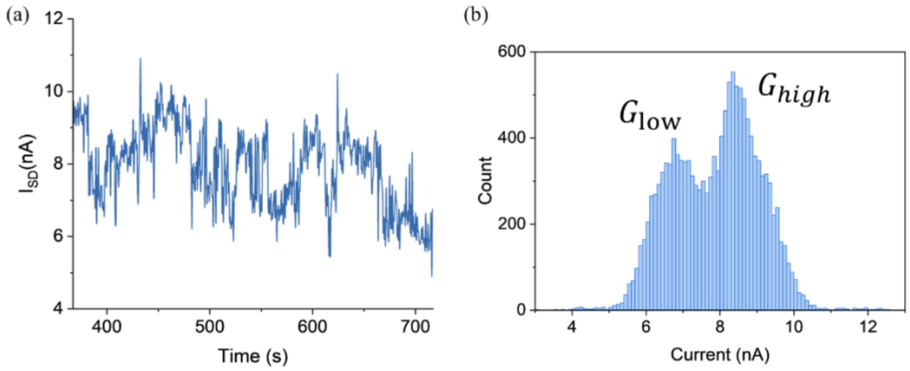

Figure S10. (a) Real-time current responses and (b) the $G_{High/Low}$ band histogram of SWCNT-FET biosensors employing toehold-mediated strand displacement (TMSD) for cortisol sensing.

The WA-SWCNT hybrid undergone TMSD process to active the aptamer and then immobilized on the chip as the FET device. Then the P-strand was re-introduced to investigate the impact of the mis-matching P strand to cortisol detection.

References

(1) Wolfe, M.; Cramer, A.; Webb, S.; Goorskey, E.; Chushak, Y.; Mirau, P.; Arroyo-Currás, N.; Chávez, J. L. Rational Approach to Optimizing Conformation-Switching Aptamers for Biosensing Applications. ACS Sens **2024**, 9 (2), 717–725. https://doi.org/10.1021/acssensors.3c02004.

(2) Integrated DNA Technologies. OligoAnalyzer^{TM} Tool. IDT DNA. https://www.idtdna.com/pages/tools/oligoanalyzer (accessed July 23, 2025).
